# Multi-drug resistant *Clostridioides difficile* isolate ST81 is prominent in hematological patients in a teaching hospital in China

**DOI:** 10.3389/fcimb.2025.1668584

**Published:** 2025-12-08

**Authors:** Qi Zhao, YaoQian Bian, XinJun Wei, Shuzhen Xiao, Zizhen Xu, Hui Li, Qingtian Li, Beiwen Wei, Jiewen Huang, Zhen Song, Yanan Zhao

**Affiliations:** 1 Department of Laboratory MedIcine, The Affiliated Yantai Yuhuangding Hospital of Qingdao University, Yantai, China; 2 Department of Laboratory Medicine, Dachang Hospital of Baoshan District of Shanghai, Shanghai, China; 3 Department of Laboratory Medicine, The Eighth People’s Hospital of Qingdao, Qindao, China; 4 Department of Laboratory Medicine, Ruijin Hospital, Shanghai Jiao Tong University School of Medicine, Shanghai, China; 5 College of Health Science and Technology, Shanghai Jiao Tong University School of Medicine, Shanghai, China

**Keywords:** *Clostridioides difficile*, hematologic diseases, genotyping, epidemiology, drug resistance

## Abstract

**Introduction:**

*Clostridioides difficile* Infection (CDI) is more prevalent in people with hematologic diseases. However, epidemiological characteristics are poorly understood.

**Methods:**

From July 2016 to November 2021, we studied the epidemiology of CDI in patients with hematological diseases at a tertiary teaching hospital in Shanghai, China.

**Results:**

In hematological patients, the prevalence of CDI was 21.6%, with 89.8% hospital-acquired infections. *C. difficile* ST81, which is a multidrug-resistant strain carrying only the toxin B, is the most common strain (38.1%), followed by ST3 (16.7%) and ST2 (9.5%). Clindamycin and moxifloxacin resistance rates of all *C. difficile* species were 64.3% and 31%, respectively, and no isolate was resistant to vancomycin, linezolid, metronidazole, teicoplanin, or daptomycin.

**Discussion:**

This study provides a comprehensive characterization of CDI in hematological patients, highlighting the urgent need for enhanced surveillance and preventive strategies against this emerging nosocomial threat.

## Introduction

*Clostridioides difficile* (*C. difficile*), a Gram-positive, obligate anaerobic bacterium, is recognized as the predominant causative agent of hospital-acquired diarrhea. Following colonization and invasion of the human gastrointestinal tract, this pathogen triggers a wide range of clinical manifestations (Aliramezani et al., 2019). *C.difficile* infection (CDI) is linked to an asymptomatic carrier state as well as severe illness, including watery diarrhea, abdominal pain and cramps, severe life-threatening fulminant colitis, and even death (Czepiel et al., 2019). Dramatically increased CDI has caused high mortality due to complications and growing economic pressures, which is posing a significant clinical challenge (Wiegand et al., 2012; Banaei et al., 2015; Lessa et al., 2015; Desai et al., 2016).

Furthermore, recurrence of infection, which occurring in up to 30% of patients after the initial episode (Gerding et al., 2015), has become a severe problem. The major virulence factors of *C. difficile*, toxin A (TcdA) and toxin B (TcdB), are encoded within a pathogenicity locus, which also includes both negative and positive regulators governing their expression (Kuehne et al., 2010). The third binary toxin (CDT) encoded by the *cdtA* and *cdtB* genes, on the other hand, is frequently found in *C. difficile* strains associated with increased CDI severity, such as ribotype (RT)027 and 078 C*. difficile* isolates (Clements et al., 2010; Persson et al., 2022). Antibiotic exposure, the elderly, weakened immune systems, and hospitalized patients have a higher risk of developing CDI, particularly in patients with inflammatory bowel disease(IBD), organ transplants, and hematological diseases (Riddle and Dubberke, 2008; Gweon et al., 2014; Zhang et al., 2016; Qin et al., 2017; Bushman et al., 2020).

Hematology patients are more easily to develop CDI due to damage of immune system, intensive chemotherapy, and extended exposure to broad-spectrum antibiotics (Gweon et al., 2014). Different research groups have reported higher incidence rates of CDI among hematological patients in comparison to other patient groups. However, previous studies mainly focusing on the risk factors that associated with the development of CDI in hematological patients and the results were often controversial (Gu et al., 2015; Dutta et al., 2021; Hung et al., 2021). In addition, *C. difficile* species differ in terms of genetic diversity, drug resistance and metabolic capacity. *C. difficile* RT027 and RT078 can use trehalose to increase infection. The most common IBD-associated lineage (Collins et al., 2018), *C. difficile* ST54, may use the host’s sorbitol and thus exacerbate clinical manifestations of IBD *in vivo* (Yang et al., 2023). However, there has been limited research on the genotyping of *C.difficile* in patients with hematologic diseases.

Therefore, we comprehensively described the epidemiology of CDI in patients with hematological diseases at a tertiary teaching hospital in Shanghai, China, from July 2016 to November 2021. The purpose of this study was to evaluate the characteristics and risk factors, molecular genotypes, antimicrobial resistance patterns to improve our comprehension of CDI in hematological patients.

## Materials and methods

### Study design and strains collection

The study was conducted in a 1, 400-bed general teaching hospital in Shanghai, China, from July 2016 to November 2021. Patients (≥18 years of age) with diagnosed acute myeloid leukaemia (AML), acute lymphoblastic leukaemia (ALL), chronic lymphocyte leukemia (CLL), chronic myeloid leukemia (CML), Hodgkin’s lymphoma (HL), and non-Hodgkin lymphoma (NHL), multiple myeloma (MM), myelodysplastic syndromes (MDS), hemophagocytic syndrome (HPS), poems syndrome, were included in the analysis. All hematological patients suspected with *Clostridioides difficile* infection (CDI) (passage of three or more unformed stools in 24 h or loose stools for more than 3 days) were tested for *C. difficile*. Glutamate dehydrogenase (GDH) assays and toxins A/B (TcdA/B) assays were performed on patient stool specimens using the C. DIFF QUIK CHEK COMPLETE immunochromatographic test kit (Alere, USA). Stools that tested positive for GDH were subsequently inoculated for 48 hours at 37 °C in an anaerobic environment with *C. difficile* CDIF selective medium (BioMerieux, France). MALDI-TOF MS was used to identify suspect isolates. *Community-acquired Clostridioides difficile infection* (CA-CDI) was defined as a CDI episode diagnosed within 72 hours of hospital admission in patients with no documented hospitalization in the preceding six months. *Hospital-acquired Clostridioides difficile infection* (HA-CDI) was defined as CDI diagnosed ≥4 days after hospital admission or CDI diagnosed within the first 72 hours of admission in patients with a documented hospitalization within the preceding six months. The collection of bacterial isolates from patient specimens and the use of related clinical information were reviewed and approved by ethics committee of Ruijin Hospital with the approval number: KY2023-083. No consent was needed for this study. Duplicate specimens from the same patients were excluded.

### Multi-locus sequence typing and toxin detection

MLST was performed according to previous publication (Griffiths et al., 2010). The genes *Adk*, *atpA*, *dxr*, *glyA*, *recA*, *sodA* and *tpi* were amplified by polymerase chain reaction (PCR) and sequenced. Results submitted to the MLST database (http://pubmlst.org/*C.difficile*) to obtain the allele profile and sequence type (ST). The evolutionary relatedness of different strains was analyzed through the online website PHYLOViZ (https://online.phyloviz.net/index) based on goeBURST algorithm and the VivaGraphJS library. After harvesting, genomic DNA was obtained and *tcdA*, *tcdB*, *cdtA* and *cdtB* were examined by a multiplex PCR, as previously described (Pituch et al., 2005).

### Antimicrobial susceptibility of *C. difficile* isolates

The minimum inhibitory concentration (MIC) of nine antibiotic, including meropenem, vancomycin, linezolid, metronidazole, moxifloxacin, teicoplanin, rifaximin, daptomycin, clindamycin, was determined by the agar dilution method, according to Clinical and Laboratory Standards Institute (CLSI) guidelines [M11-A8]. ATCC 70057 was used as control. The breakpoints for meropenem, metronidazole, moxifloxacin, clindamycin were based on CLSI recommendations for anaerobes[M100-S35]. The vancomycin resistance breakpoint (MIC >2 mg/L) was defined according to the European Committee on Antimicrobial Susceptibility Testing (EUCAST). The breakpoints for linezolid and rifaximin were based on published literature (Marin et al., 2015; Schwanbeck et al., 2025).

### Whole-genome sequencing and analysis

The Gentra Purgene Yeast/Bact. Kit was used to harvest genomic DNA from *C. difficile* overnight cultures(Qiagen, Germany). The genome sequence of *C. difficile* was sequenced by Shanghai Majorbio Bio-Pharm Technology Co., Ltd. (Shanghai, China) according to Illumina instructions The SOAPdenove v2.04 tool (with GapCloser v1.12) was used to assemble the readings (http://soap.genomics.org.cn/). The ST81 cluster genomes were constructed and analyzed using CLC Genomics Workbench, with *C. difficile* M68 (FN668375.1) as a reference.

### Statistical analysis

Statistical analyses were performed using software, SPSS Version 27.0 (SPSS, USA). Either the two-tailed unpaired Student’s t-test or the Mann–Whitney U-test was used for comparisons of continuous variables with or without a normal distribution. Chi-square were used to compare categorical variables if differences existed among groups. A *P* value of less than 0.05 was considered statistically significant.

## Results

### Prevalence and characteristics of CDI in hematological patients

Between July 2016 and November 2021, 227 diarrheal patients with hematological diseases were sampled in a teaching hospital. As shown in **Table 1**, the investigation included 53 (23.3%) patients with acute leukaemia, 93 (41.0%) patients with multiple myeloma (MM), 65 (28.6%) patients with non-lymphoma Hodgkin’s (NHL), and 16 (7.0%) patients with other hematological diseases. Among the participants, CDI was diagnosed in 49(21.6%) patients, with a mean age of 56.9 years and 53.1% (n=26) females. The prevalence for HA-CDI was 89.8% (44/49) and 10.2% (5/49) for CA-CDI (Not shown). Recurrence is a huge threat to CDI patients. The prevalence of recurrence was 8.1% (4/49) and ST81 was the causative strain in three of the four recurrence cases. CDI was more common in patients with acute leukemia, but the difference was not statistically significant (32.1% vs 16.1%, 20.0% and 25.0%, *p* = 0.152). Interestingly, 89.8% of CDI patients presented with at least one co-infectious disease. Furthermore, our analysis revealed that hematologic patients with a prior CDI within one year exhibited a significantly higher infection rate compared to those without prior infection (10.2% vs. 0%, *p* < 0.001). In addition, haemoglobin levels were lower in CDI patients than in the non-CDI group (74.8 ± 26.2 vs 84.0 ± 23.3, *p* = 0.020). There was no difference between the CDI+ and CDI- groups in other laboratory parameters such as white blood cell count, neutropenia, platelet count, albumin level, and serum creatinine level.

**Table 1:** Clinical characteristics of *C.difficile* infection in hematological patients.

Characteristics	All	CDI+	CDI-	*p*
Number of subjects, N (%)	227	49 (21.6)	178 (78.4)	0.152^a^
acute leukemia	53	17 (32.1)	36 (67.9)	
multiple myeloma	93	15 (16.1)	78 (83.9)	
non-Hodgkin’s lymphoma	65	13 (20.0)	52 (80)	
Others	16	4 (25.0)	12 (75.0)	
Gender, N (%)				
Female	110 (48.5%)	26 (53.1%)	84 (47.2%)	
Age group, N (%)				
<60 years-old	113 (49.8%)	24 (49.0%)	89 (50.0%)	0.899 ^a^
≥60 years-old	114 (50.2%)	25 (51.0%)	89 (50.0%)	
Age (years, x ± SD)	57.8 ± 12.5	56.9 ± 12.7	58.1 ± 12.5	
White blood cell count (10^9^/L), mean ± SD	4.9 ± 6.3	4.8 ± 8.4	4.9 ± 5.6	0.468 ^b^
Neutropenia (<0.5*10^9^/L)	73 (32.2%)	17 (34.7%)	56 (31.5%)	0.668 ^a^
Hypoalbuminemia (<35g/L)	133 (58.6%)	29 (59.2%)	104 (58.4%)	0.151 ^a^
Platelet count (10^9^/L), mean ± SD	100.1 ± 103.0	84.7 ± 101.6	104.1 ± 103.2	0.143 ^b^
Haemoglobin (g/L), mean ± SD	82.1 ± 24.2	74.8 ± 26.2	84.0 ± 23.3	0.020 ^b^
Albumin level(g/L), mean ± SD	33.4 ± 5.9	33.9 ± 5.3	33.27 ± 6.0	0.640 ^b^
Serum creatinine level (umol/L), mean ± SD	83.0 ± 117.5	75.53 ± 126.4	85.0 ± 112.3	0.105 ^b^
Occurrence of co-infection, N (%)				
0	72 (31.7)	5 (10.2)	67 (37.6)	P<0.0001
1-3	155 (68.3)	44 (89.8)	111 (62.4)	
CDI within one year before diarrhea, N (%)	5 (2.2%)	5 (10.2%)	0 (0.0%)	0.0001 ^a^

Data are presented as N (%) or mean ± SD, ^a^Chi-square, ^b^Mann–Whitney U-test.

CDI+, *C. difficile* infection patients; CDI-, *C. difficile* negative patient.

### Microbiological characters of CDI patients

**Figure 1:**
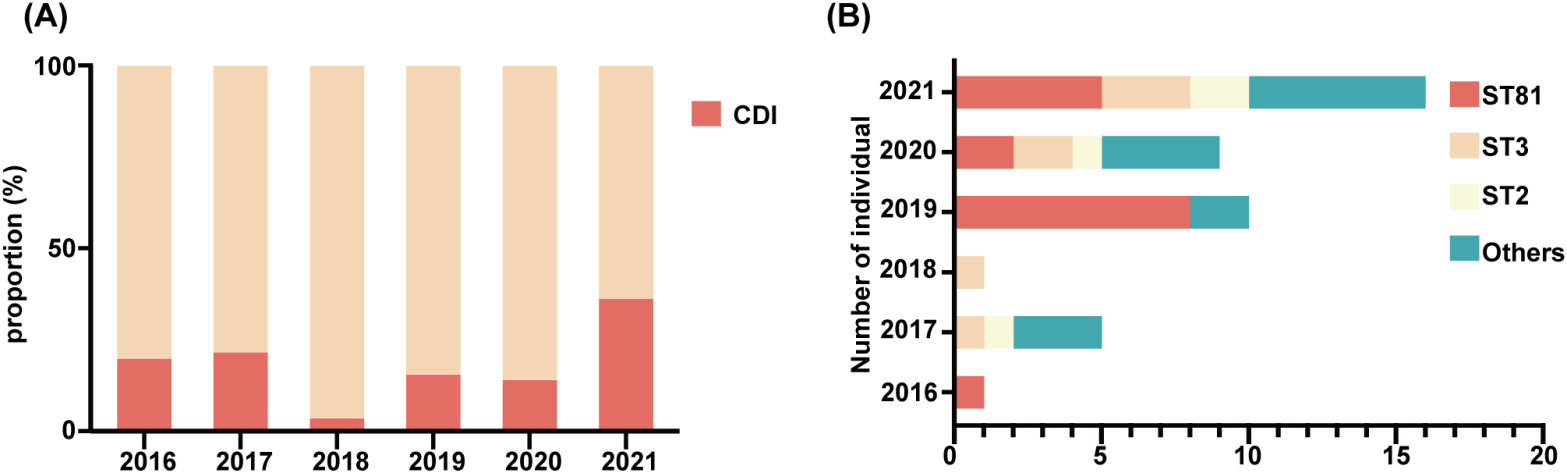
Detection and distribution of *C*. *difficile* subtypes from 2016 to 2021. **(A)** Dynamic changes in proportion of the CDI from 2016 to 2021. **(B)** Distribution of different genotypes per year, genotypes containing only one isolate were uniformly classified as “Others”.

**Figure 2:**
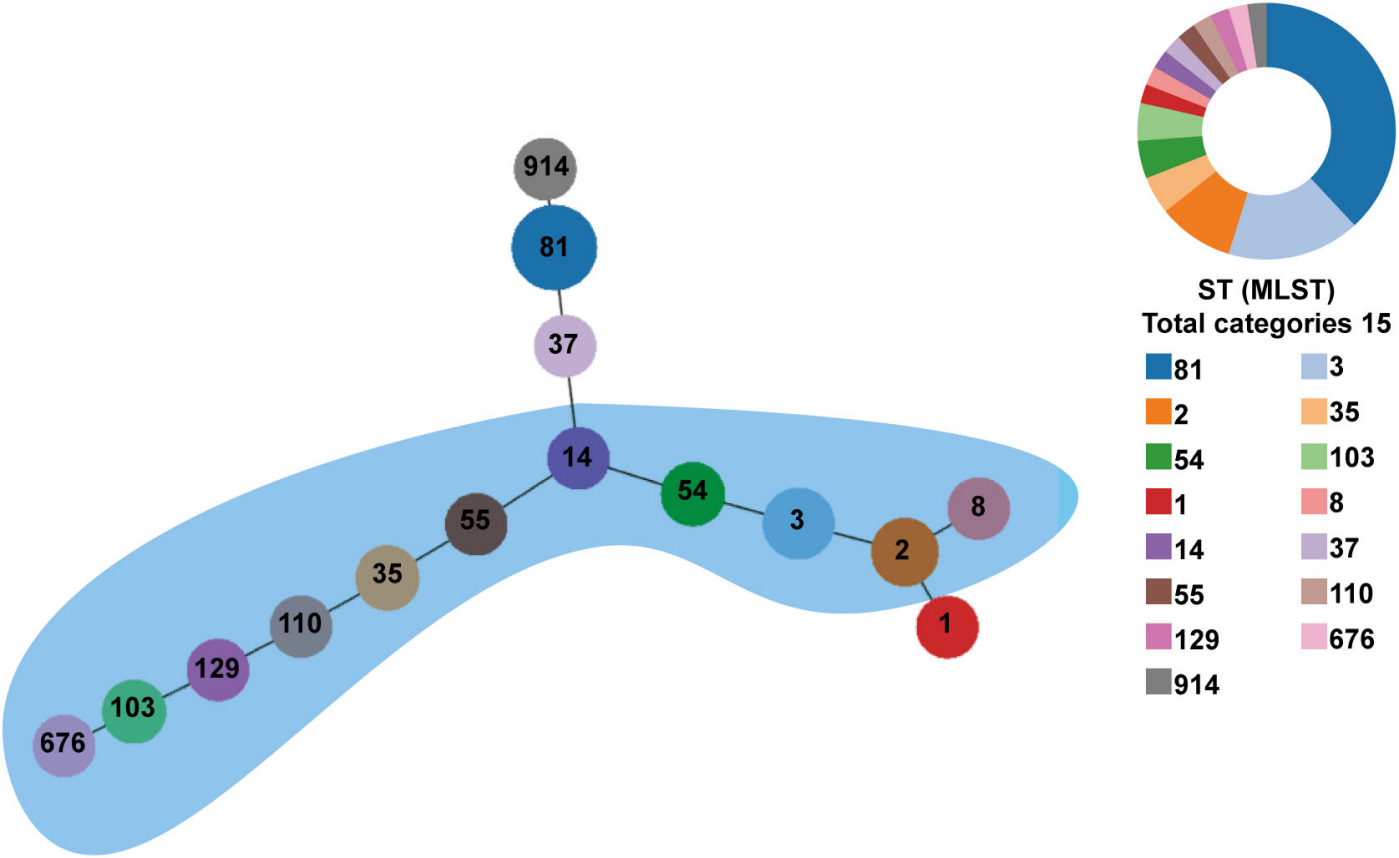
Phylogenetic tree of the *C. difficile* strains from Hematological Patients. PHYLOVIZ analysis showing the genetic relationship among 42 isolates collect from the hematological patients in this study. MLST was presented in different colors. The node size is proportional to the quantity of isolates. The links represent the relationship between the two STs. Clade 1 is clustered in a blue area.

Of 49 toxin-positive CDI cases, 42 underwent microbial analysis, while seven culture-negative cases were excluded. Since toxin detection confirmed CDI diagnosis, culture failure in these seven cases may reflect prior antibiotic use or non-viable bacteria. As shown in **Figure 1A**, from 2018 to 2021, the detection rate of *C. difficile* increased year after year. All isolates were defined into 15 STs, with ST81 (38.1%, 16/42) being the most common clone identified, followed by ST3 (16.7%; 7/42), and ST2 (9.5%; 4/42) as illustrated in **Table 2** and **Figure 1B**. The widely epidemic ST54, has a very low detection rate (n=2) as well as ST37(n=1), being considered as responsible for outbreak in China, Europe, and North America (Luo et al., 2018; Li et al., 2019). The phylogenetic relationships of the 42 isolates were analyzed using an online website based on the ST patterns, as shown in **Figure 2**. Three major clonal complexes (CCs) were identified, with clade 1 being the most common in this study. ST81, the most common clone, was classified as clade 4 (**Table 2**).

**Table 2:** Genotypes and toxin of *C. difficile* isolated from hematological patients.

Toxin genotypes	STs	MLST clade	Number	Rate (%)
A-B+CDT-	/	/	19	45.3
	ST81	4	16	38.1
	ST37	4	1	2.4
	ST676	4	1	2.4
	ST914	4	1	2.4
A+B+CDT-	/	/	22	52.3
	ST3	1	7	16.7
	ST2	1	4	9.5
	ST35	1	2	4.7
	ST54	1	2	4.7
	ST103	1	2	4.7
	ST110	1	1	2.4
	ST14	1	1	2.4
	ST8	1	1	2.4
	ST55	1	1	2.4
	ST129	1	1	2.4
A+B+CDT+	/	/	1	2.4
	ST1	2	1	2.4
Total	/	/	42	100

A +B+: toxin A-positive, toxin B-positive strain; A-B +: toxin A-negative, toxin B-positive strain.

Four toxin genes (*tcdA, tcdB, cdtA*, and *cdtB*) were analyzed in our study. The majority of isolates (52.3%, 22/42) were positive for both *tcdA* and *tcdB* genes, while 45.3% (19/42) were negative for *tcdA* but positive for and *tcdB* genes. All ST81, the most common strains, carried only the toxin B gene. A ST1 isolate carrying four toxin genes (*tcdA*, *tcdB*, *cdtA*, and *cdtB*) was identified, representing the only strain in our study capable of producing binary toxin.

### Transmission of *C.difficile* ST81

**Figure 3:**
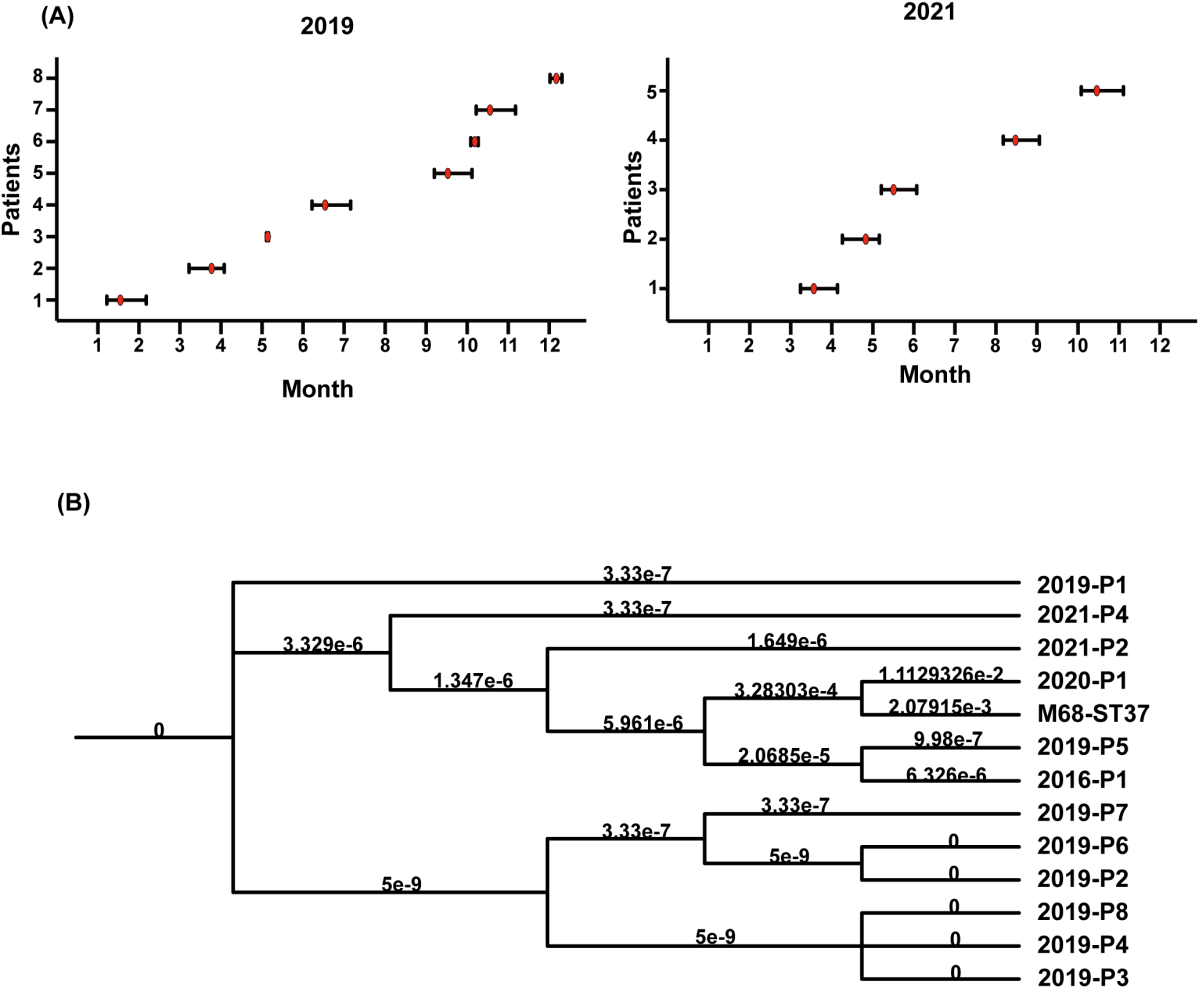
Temporal graph of 13 isolates of ST81 isolated in 2019 and 2021 and ST81 spread in Jan. and June 2019. **(A)** Temporal graph of 8 ST81 in 2019 and 5 ST81 in 2021. Line segments reflected the length of hospital stays and red dot represented detection time. **(B)** Analysis of phylogenetic tree within *C*. *difficile* genotype ST81. The reference genome was M68-ST37.

Interestingly, combining **Figures 1A, B**, ST81 was revealed to be a contributor of the increase in CDI in 2019 (8/11 isolates), whereas 2021 isolates exhibited greater genetic diversity. Thus, we perform an epidemiological investigation to show spatial and temporal clustering of ST81 cases. Patient 2019-P1 (Bed 25) and 2019-P7 (Bed 03)was housed in two separate rooms from other cases. Furthermore, 2019-P5 and 2019-P8 (Beds 10 and 12, same ward) had a two-month interval between admissions (**Figures 3A, B**). In contrast, patients 2019-P2, 2019-P3, and 2019-P4 (Beds 17, 13, and 13, respectively) shared the same ward. Notably, 2019-P3 developed CDI following May 2019 hospitalization; one month later, 2019-P4 was admitted to the same bed (Bed 13) and contracted an ST81 strain. Phylogenomic analysis demonstrated that these isolates shared high genetic relatedness (**Figure 3B**), indicating that they were extremely similar, suggest probable transmission. Although 2019-P6 (Bed 19) was admitted >6 months later after 2019-P2 (Bed 17), since their proximity in adjacent rooms and phylogenetic clustering of the isolates suggest potential environmental persistence or undetected transmission. Collectively, those may indicate that ST81 was spreading in patients with hematologic disease.

### Antimicrobial resistance

**Figure 4:**
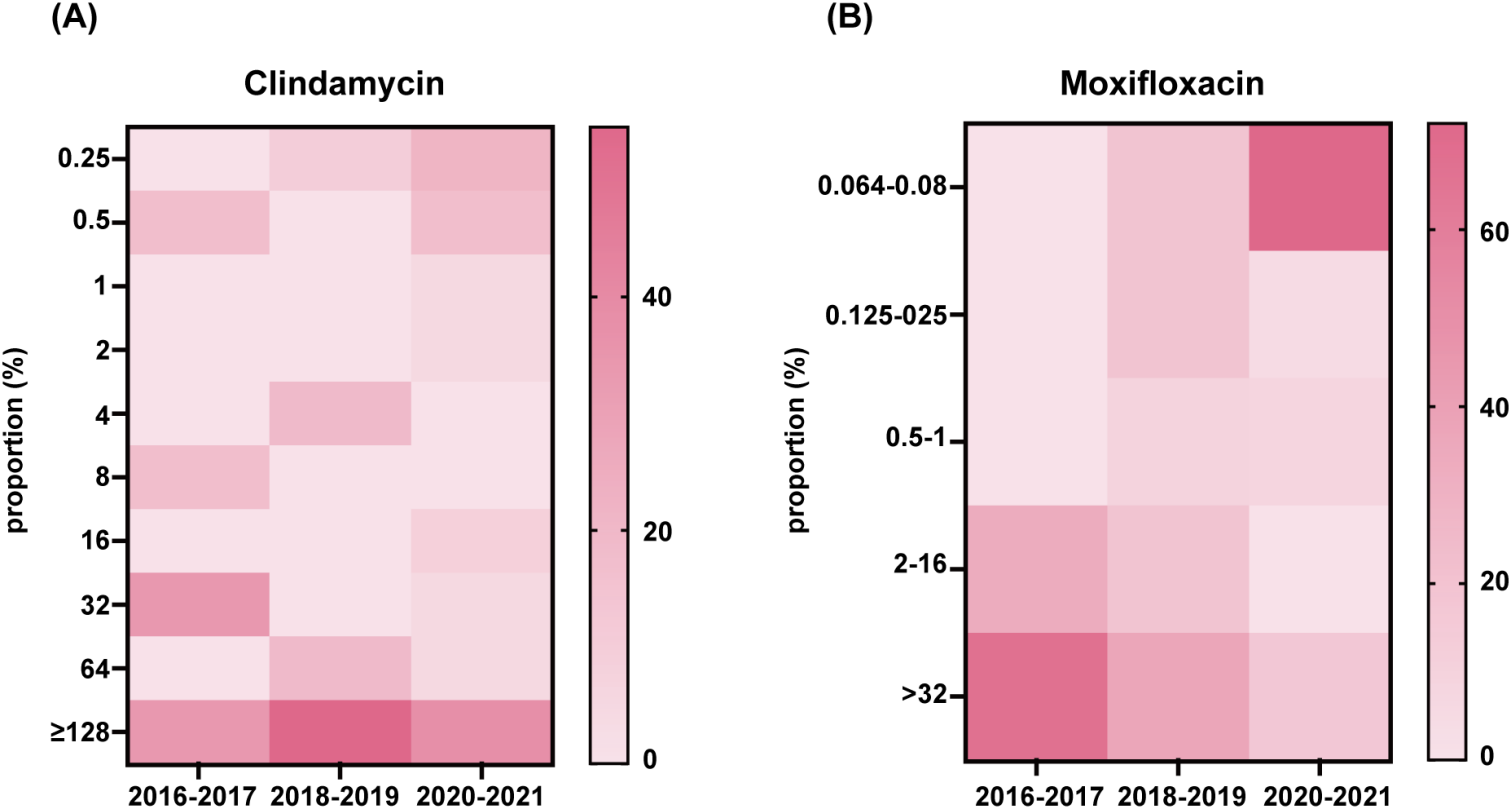
Clindamycin and moxifloxacin MIC distributions in 42 isolates. Distribution of the MIC to antibiotic clindamycin **(A)** and moxifloxacin **(B)** of all isolates was presented.

All *C.difficile* isolates were evaluated for antimicrobial resistance to nine antibiotics (meropenem, vancomycin, linezolid, metronidazole, moxifloxacin, teicoplanin, rifaximin, daptomycin, and clindamycin). **Table 3** shows the antibiotic resistance patterns of all *C. difficile* strains. 64.3% of the strains were resistant to clindamycin, and high degree of resistance persisted from 2016 to 2021 (MIC≥32 mg/L), with a MIC50 = 32 mg/L and a MIC90 = 128 mg/L (**Figure 4A**). Resistance to moxifloxacin was 31%, with a decreasing trend (**Figure 4B**). Meropenem and rifaximin had resistance rates of 9.5% and 7.1%, respectively. No isolate showed positive for resistance to vancomycin, linezolid, metronidazole, teicoplanin, or daptomycin. The dominant strains ST81 were more resistant than non-ST81 strains (*p* = 0.05). More than half of them had at least two drug resistances, with 31.3% having two and 18.8% having three. ST81 had a much higher rate of resistance to moxifloxacin than the other STs (50% VS 19.2% *p* = 0.036). No statistically significant differences in resistance rates were observed between ST81 and other STs for meropenem (6.3% VS 2.7%), rifaximin (12.5% vs 0), or clindamycin (75% VS 53.8%) (all *p*>0.05).

**Table 3:** Antimicrobial susceptibility profiles of *C. difficile* isolates (MIC50, MIC90, and MIC range).

Antimicrobial agent (break point mg/L)	All strains (n = 42)			Resistant (%)
	MIC_50_ (mg/L)	MIC_90_ (mg/L)	MIC range (mg/L)	
Clindamycin (R ≥ 8) ^a^	32	≥128	0. 25-≥128	64.3
Moxifloxacin (R ≥ 8) ^a^	0.125	≥32	0.064-≥32	31
Meropenem (R ≥ 16) ^a^	3	6	0.125-≥32	9.5
Rifaximin (R ≥ 32) ^c^	≤0. 064	≤0. 064	≤0. 064-≥32	7.1
Vancomycin (R ≥ 2) ^b^	0.25	0.64	0.016-2	0
Linezolid (R ≥ 4) ^c^	0.5	1.5	0.016-1.5	0
Metronidazole (R ≥ 32) ^a^	0.064	0.25	0.016-4	0
Teicoplanin (NA)	0.064	0.25	0.016-0.5	0
Daptomycin (NA)	≤0. 064	≤0. 064	≤0. 064-1	0

a Resistance breakpoints according to Clinical and Laboratory Standards Institute (CLSI) guidelines (M100-S35).

b Resistance breakpoints according to European Committee on Antimicrobial Susceptibility Testing (EUCAST).

c Resistance breakpoints according to published literature.

## Discussion

*Clostridioides difficile* Infection (CDI) has been more common in recent years, and it is particularly prevalent in individuals with hematological illnesses, particularly those with hematological malignancies. A meta-analysis of 50 publications from 14 Chinese provinces found that the pooled prevalence of CDI in China was 11.4% (2696/26, 852) (Wen et al., 2023). In contrast, our 6-years study revealed a significantly higher CDI detection rate (21.6%) among patients with hematologic disorders, representing approximately twice the general population. These findings underscore the urgent need for targeted clinical strategies to address CDI management in this high-risk cohort.

According to molecular epidemiology studies *C. difficile* has distinct characteristics and genotypes in different areas or people. The ST1/RT027 lineage is widespread in Europe and North America, but the RT017 lineage, which includes ST37 and ST81, is abundant in Asia (Clements et al., 2010; Arvand and Bettge-Weller, 2016; Aptekorz et al., 2017; Imwattana et al., 2020). ST54 is more prevalent in people with inflammatory bowel disease (Yang et al., 2023). MLST analysis in our study identified 15 distinct sequence types (STs), with ST81 representing the predominant genotype (38.1%), followed by ST3 (16.7%) and ST2 (9.5%). The predominance of ST81 is consistent with recent epidemiological trends in China that ST81 has replacing the earlier dominance of ST54 and ST37 strains become the predominant circulating genotype (Cheng et al., 2020; Yang et al., 2020; Xia et al., 2024), suggesting that hematologic patients share similar genotype distributions with general populations. ST54, which is commonly epidemic, has an extremely low detection rate, as does ST37. One highly virulent *C. difficile* ST1/RT027 strain was found in our study. ST81 was not only the most common, but also the most resistant, with 75% demonstrating resistance to at least one antibiotic. Three of the four recurring cases was ST81. In 2019, whole-genome sequencing revealed that ST81 was transmitted in at least two wards. Multi-antibiotic resistance may make it simpler to be screened out and overgrow in the intestinal tract, developing CDI. Furthermore, it has been revealed that ST81 has a greater ability to form spores, which may contribute to its persistence in the external environment, transmission or recurrence among patients (Su et al., 2021). Therefore, ST81 should be of special concern in people suffering from hematological diseases. We strongly advocate for implementing molecular typing-based active surveillance of CDI, with particular emphasis on monitoring the hypervirulent ST81 clone, in hematology units. This approach should be prioritized as a critical strategy for future infection control.

This research has some limitations. For starters, the population is modest. Despite the fact that the trial ran from July 2016 to November 2021, just 227 patients were included. Second, bias is unavoidable in retrospective studies, and causality cannot be clearly deduced.

## Data Availability

The sequencing data reported in this study have been deposited in the NCBI Sequence Read Archive (SRA) under BioProject accession PRJNA1370298.
